# Effect of data self-collection as an activating teaching method in a statistical software course in medical biometry – a pilot study

**DOI:** 10.3205/zma001156

**Published:** 2018-02-15

**Authors:** Benjamin Mayer, Ulrike Braisch, Marianne Meule, Andreas Allgoewer, Silvia Richter, Rainer Muche

**Affiliations:** 1 Ulm University, Institute for Epidemiology and Medical Biometry, Ulm, Germany

**Keywords:** Activation, biostatistics, software, SPSS

## Abstract

**Background: **Biostatistics is an integral part of the studies of human medicine. Students learn the basics of analyzing and interpreting study results. It is important to demonstrate the subject’s relevance by means of appropriate measures to maximize learning success. We investigated whether an active involvement of students in the process of data collection may improve test performance and motivation among medical students.

**Methods:** We conducted a pilot study comparing active involvement of students (n1=45) in the process of data collection and standard education (n2=26). All students of this pilot study participated in an observational study assessing their preferences regarding sweets or salty munchies, and students of the experimental group subsequently used this data set during the exercises throughout the semester. Primary and secondary endpoints were examination success and motivation respectively.

**Results:** Superiority of the activating teaching method could not be demonstrated (intervention: 109.0 points (SD 8.8), control: 113.8 points (SD 6.5)). The course ratings were superior in the intervention group (median grade 1 vs. median grade 2 in the control group), although this was not a significant improvement (p=0.487).

**Conclusions: **Biostatistics education should incorporate approaches contributing to a better understanding of learning contents. Possible reasons why this pilot study failed to prove superiority of the intervention were a lack of sample size as well as the good grades in the control group. The presented teaching concept has to be evaluated by means of a larger sample enabling more valid conclusions. Furthermore, the considered research question in the experimental group may be changed to a more relevant one for medical practice.

## Zusammenfassung

**Hintergrund: **Die Medizinische Biometrie ist ein zentraler Bestandteil des Studiums der Humanmedizin. Die Studierenden lernen die Grundlagen der Analyse und Interpretation von Studienergebnissen. Die Relevanz des Fachgebietes sollte durch die Anwendung geeigneter Lehrmaßnahmen veranschaulicht werden, um den Lernerfolg zu maximieren. Wir untersuchten, ob eine aktive Beteiligung der Studierenden im Rahmen des Datenerhebungsprozesses den Erfolg im Testat und die Motivation bei Studierenden der Humanmedizin erhöhen kann.

**Methoden: **Wir führten eine Pilotstudie durch, bei der eine aktive Beteiligung von Studierenden im Datenerhebungsprozess (n1=45) verglichen wurde mit einem Standardansatz der Lehre (n2=26). Alle Studierenden dieser Pilotstudie nahmen Teil an einer Beobachtungsstudie, in deren Rahmen die Vorliebe für Süßigkeiten oder salzige Knabbereien erhoben wurde. Die Studierenden der Interventionsgruppe arbeiteten anschließend während der Seminarübungen des gesamten Semesters mit diesem Datensatz. Die primären und sekundären Endpunkte der Studie waren der Erfolg im Testat sowie die Motivation.

**Ergebnisse: **Eine Überlegenheit der aktivierenden Lehrmethode konnte nicht gezeigt werden (Interventionsgruppe: 109.0 Punkte (SD 8.8), Kontrollgruppe: 113.8 Punkte (SD 6.5)). Die Bewertungen des Seminars waren in der Interventionsgruppe leicht überlegen (mediane Note 1 vs. mediane Note 2 in der Kontrollgruppe), jedoch stellte dies keine signifikante Verbesserung dar (p=0.487).

**Schlussfolgerungen:** Die Lehre im Fach Medizinische Biometrie sollte didaktische Ansätze integrieren, die zu einem besseren Verständnis der Lehrinhalte beitragen können. Mögliche Gründe, weshalb eine Überlegenheit des betrachteten Lehrkonzeptes hier nicht gezeigt werden konnte, könnten eine zu geringe Fallzahl sowie die guten Bewertungen der Kontrollgruppe gewesen sein. Das vorgestellte Lehrkonzept sollte auf der Basis einer größeren Stichprobe evaluiert werden, um zu valideren Schlussfolgerungen zu gelangen. Zudem sollte die zugrunde gelegte Forschungsfrage in der Interventionsgruppe geändert werden in eine für die medizinische Praxis relevantere.

## Introduction

Biostatistics is an integral part of the studies of human medicine. In Germany, the subject shows up in the curriculum in combination with epidemiology and medical informatics. This so-called cross-sectional subject Q1 is highly important for prospective physicians, since it introduces the basics of planning, conducting, analyzing, interpreting, and reporting studies in medical research. Biostatistics is especially geared to teach the statistical-mathematical principles of standard approaches of data analysis. Teaching epidemiology is primarily focused on essential aspects of observational studies, which are largely applied to investigate the development and dissemination of diseases. Ultimately, the interest of medical informatics is on the appropriate use of information technology methods (e.g. databases or statistical software) in order to efficiently manage and analyze medical data sets. 

The overriding educational objective of lectures in biostatistics for students of human medicine is to familiarize them with the basic knowledge and terminology of medical statistics. This is essential for an autonomous preparation of scientific work (e.g. dissertation or research article). Moreover, students should be able to understand the content of published articles in order to evaluate their importance appropriately. This is an important skill for the subsequent professional life, no matter in which field of medical research [1]. Of course, physicians who are primarily involved in research projects have to cope with published research articles more often, but also practical physicians should be able to profoundly assess the significance of research findings. 

Despite the undeniable importance of biostatistics in practice, it seems that the subject does not enjoy great popularity among students compared to other, more clinical subjects. The main reasons for that may be a general unpopularity of mathematical disciplines and an insufficient connection to practical relevance. Therefore, it is all the more important to incorporate descriptive examples in the lectures in order to maximize learning success. Ideally, activating teaching methods should be additionally applied. It has been demonstrated that this didactic approach could improve learning success [2], [3]. Also, from the motivational model of Keller and Kopp (*ARCS- attention, relevance, confidence, satisfaction*) it could be derived that successful didactical approaches necessarily have to include specific motivational components [4]. 

A first step towards more practical relevance has been realized more than ten years ago at our institute. A PC-based seminar in biostatistics has been designed and implemented. Since then a maximum of 76 students per semester (25% of the full cohort) can work on practical exercises in the field of medical statistics [5]. The analyses base on real data from a finalized study in pediatrics. This observational study investigated the prevalence of type 2 diabetes mellitus in obese children and adolescence [6]. By means of this dataset students work on exercises in descriptive statistics, confidence intervals, event time analysis, correlation and regression analysis, and statistical tests. Initially, SAS Analyst was used for the analysis, but in the meantime we switched to the statistical software SPSS. This software is not only used for the exercises, but also for processing the short examinations at the end of each seminar. Students have to generate the results using SPSS and afterwards all solutions are recorded in Microsoft Access input screens. Correction and marking are subsequently done automatically by means of respective SAS programs. The results of all short examinations are cumulated and replace the common exam at the end of the semester. 

This initial approach of a PC-based seminar in biostatistics improved the practical relevance [7]. However, due to limited capacity in the PC-labs it is still not possible for the majority of the students to attend the PC-seminar. They have to attend the regular seminar where the exercises and short examinations are handled using pocket calculators. This is of course not appropriate and unconducive regarding the goal of arousing interest for the subject. The increased practical relevance achieved by implementing the PC-seminar could be further enhanced if the students were actively involved in the process of data collection. This would enable students to subsequently work on exercises which base on their own data. Thus, self-assessment of research data is in the sense of integrating activating teaching methods which are evidently able to increase learning success [2], [8], [9]. Consequently, we developed our idea of the study concept presented in the following. 

In the course of diverse university events (e.g. anniversary of Ulm University, Pupils’ University, adult training courses) we presented an example study in order to demonstrate the principles of biostatistics to a broad and non-specialist audience. Interested visitors were able to participate in this observational study which captured primarily the participants’ preference for sweets (in German so called “Naschkatzen”) or salty munchies (“Nagetiere”). In addition, few basic variables like age, gender, body height and body weights were collected. The analysis of this observational data primarily focused an evaluation of possible differences between the two groups regarding the participants’ body mass index (BMI) [10]. The acronym NaNa was used for this example study because of the afore mentioned German labels of both study groups. 

Consequently, the NaNa concept provided a promising opportunity regarding the aim of raising practical relevance in teaching biostatistics. On the one hand, students would be involved actively in the data collection process. Thus, basic principles of data collection as well as the associated problems can be discussed while implementing a respective data base. On the other hand, motivation may be increased by the fact that students will work on data which they have collected on their own. This may have positive effects on learning success. Altogether, it seemed possible in this way to emphasize the relevance of biostatistics. To evaluate the effect of this interactive didactic method we conducted a prospective, controlled, two-arm, single center, pilot study with observational data. Specifically, the NaNa-based teaching concept was compared to the current standard of education in the statistical software course in biostatistics. Our research hypothesis was that the NaNa concept is superior with respect to learning success and motivation. 

The article is structured as follows: first, the applied didactic approaches (intervention and control) are described along with the collected study variables. Afterwards, the results of both research hypotheses, improvement of learning success and motivation, are presented. The article concludes with a discussion of the takeaways regarding the effect of the didactic intervention as well as the potential of the NaNa concept to be established as a new standard for the statistical software course. 

## Material and methods

### Study design, participants and didactic intervention

The study followed a mono-centric, two-arm, controlled design with prospective observational data. There was no formal sample size calculation because of the pilot-character of this study, although the sample size could have calculated based on information about the primary outcome (examination success) in the control group. Instead, all students of human medicine at Ulm University who attended the PC-based seminar on biostatistics during the winter term 2016/17 participated in our study (*N*=71). The students belonged to one of four seminar groups (maximum space for 24 and 14 students, respectively) which were instructed by three lecturers. Nearly all students (*N_dataset_*=70) attended data collection in the course of an unscheduled date before the official launch of the seminar. The two larger seminar groups (*n_1_*=45) received the NaNa concept as the leading didactic intervention and worked with the self-assessed participants data during the semester. The two smaller groups (*n_2_*=26) served as a control intervention and worked with real observational data of a finalized study in pediatrics [6]. Hence, the control group received the standard didactic intervention which has been used for more than 10 years in the respective statistics software course. Students of both interventional groups processed exactly the same exercises during the tutorials and examinations, respectively. Only the variables used in the exercises differed. All students gave written consent to participate in the study. The ethics committee of Ulm University approved the study. 

#### Data assessment

Data assessment was pseudonymized. Available study participant (*N*=70) gave information about demographic and health-related variables. Furthermore, variables describing the consumption behavior of the offered snacks were assessed, in particular type of snack (sweets or salty munchies), frequency of snack consumption and main reason for the consumption. There was a wide choice of both offered snack groups in order to cover all preferences. 

Relevant demographic variables included age, gender, body height, body weight, and origin. Health-related variables involved physical activity, blood pressure, smoking status, chronic diseases, and allergies. The collected data were used to create exercises for the tutorials and examinations, respectively. All exercises were directly guided by those of the standard didactic intervention. 

#### Outcomes

The primary objective for an assessment of the didactic intervention’s effect was the cumulated sum of points from all single examinations during the semester and the accompanied grades, respectively. The results of the lecture’s evaluation by the students was a secondary objective of the trial. 

#### Statistical analysis

First, a comparison of both collectives regarding demographic variables was conducted descriptively. Continuous variables were described using mean, standard deviation (SD), median, and quartiles (where appropriate). Frequencies were calculated for categorical variables and the chi-square test or Fisher’s exact test were used, respectively, to compare them subsequently. The unpaired t-test was used to analyze the primary endpoint. From earlier semesters it could be expected that the cumulative sum of points will be normally distributed. The Mann-Whitney-U test was used for the evaluation of the secondary endpoint (acceptance) due to its ordinal scale level. Cohen’s effect size (*d*) has been additionally calculated in order to express the strength of the effect sizes [11]. A p≤0.05 was considered significant, whereas all results were interpreted in an explorative manner. Only single missing values occurred in the final data set, whereas no missing values were present in variables concerning both the primary and secondary outcome. The statistical software R (version 3.2.1, http://www.r-project.org) was used for the analyses. 

## Results

Study participants showed no difference compared to those who did not attend the PC-based seminar in biostatistics during the winter term 2016/17 with respect to the distribution of age and gender. On average, they were 23.9 years old (SD 2.9) and 43% were male (see Table 1 (Tab. 1)). The majority attended the 6^th^ or 7^th^ semester (77%) and decided to take sweets (76%) in the course of data collection. Independent of their choice for either snack groups 80% stated “enjoyment” as the main reason for the consumption. There was no difference between the intervention and control groups regarding age and gender, but with respect to the distribution of semesters (p=0.0003).

**Figure 1:**
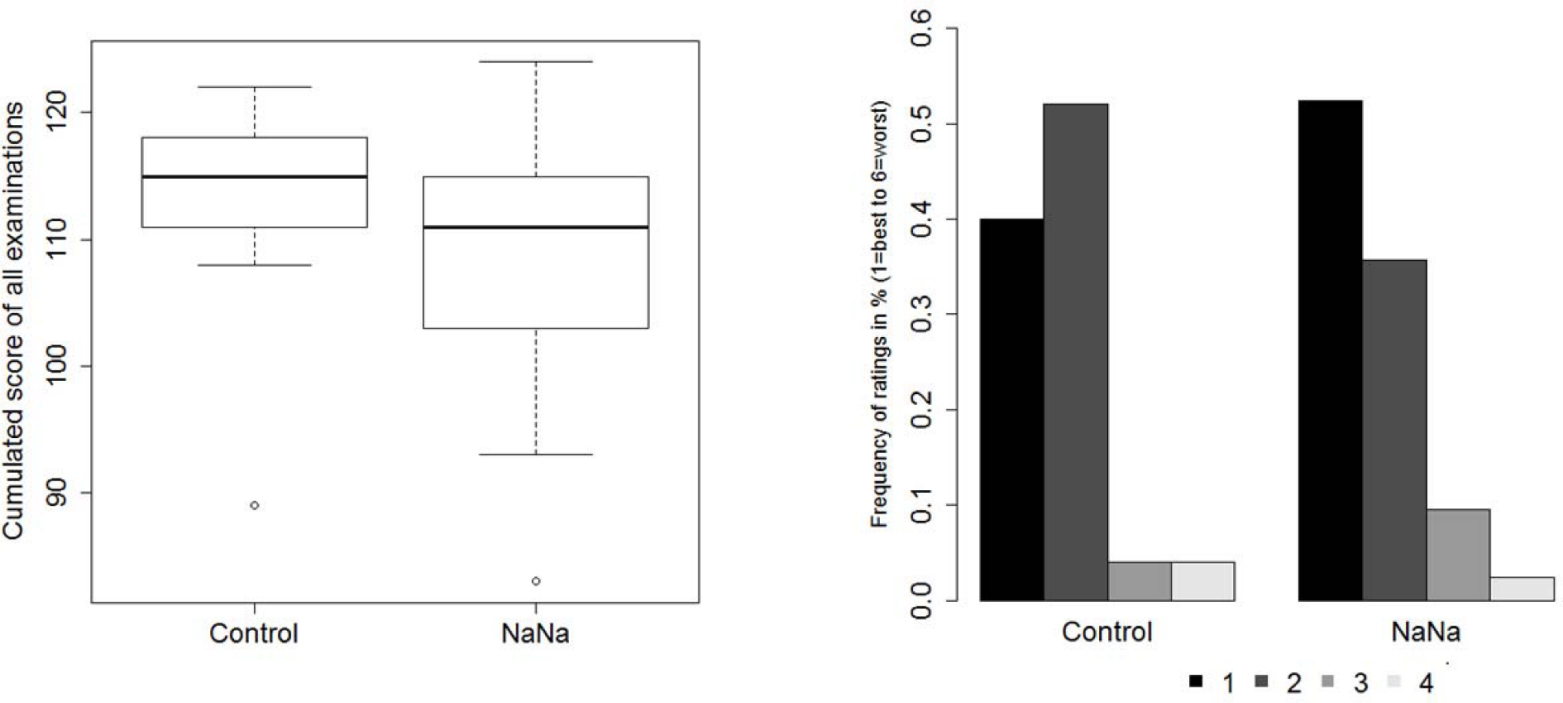
Comparison of study groups with respect to attained sum of points (left) and evaluation (grades 1-6) of the seminar (right)

The primary research hypothesis of a superiority of the NaNa concept with respect to improved learning success and motivation could not be demonstrated by means of the collected study data. On the contrary, students of the control group were even slightly superior with a mean of 113.8 points (SD 6.5) from all examinations compared to 109.0 points (SD 8.8) on average for students of the NaNa group (p=0.012, see Figure 1 (Fig. 1)). This corresponds to an effect size of *d*=0.62 indicating a moderate effect. Of course, this was also confirmed by an evaluation of the resulting grades (1.6 (SD 0.6) in the control group vs. 2.1 (SD 0.7) in the NaNa group, i.e. *d*=0.77, p=0.001). Nevertheless, the interventional group scored the seminar slightly better (median score 1) than the control group (median score 2), whereas the scoring based on regular school grades from 1=best to 6=worst. This difference was not statistically significant (*d*=0.16, p=0.487). The same tendency was also observed when looking at more detailed questions referring specific aspects of the lecture evaluation. The median score for “I would attend the lecture again.“ was 6 (“complete agreement“) in the NaNa group and 5 (“agreement“) in the control group. The question “I have learned a lot” was scored equally in both groups with a median of 5 (“agreement”). 

## Discussion

All subjects in the curriculum of the studies of human medicine which are not primarily focused on clinics, as e.g. biostatistics, have to deal with the same problems. Often missing practical relevance is mentioned. Since many of these subjects are naturally focused on fundamentals (e.g. biochemistry, physics) or methodological aspects, education in these fields should comprise practical elements. These may include innovative technical devices, e.g. virtual reality glasses in cardiology, patient dummies with integrated measurement sensors in emergency medicine, or the application of didactic apps which support the students [12], [13]. Alternatively, activating teaching methods empirically lead to a better understanding of the teaching contents [2], [3]. Our NaNa learning approach explicitly addressed the attention and satisfaction aspects of Keller and Kopp’s ARCS model [4]. Also the confidence aspect was considered by means of the repeated examinations (self-control) during the semester. However, the relevance aspect may not be perfectly incorporated in our study. Students in the standard educational group worked on a data example (prevalence of type 2 diabetes mellitus) of higher medical relevance compared to students of the experimental group (differences between students preferring sweets instead of salty munchies). 

Lectures in the field of medical statistics should interconnect the theoretical aspects of study planning, data analysis and interpretation to practice-oriented research questions, data and instruments, respectively, to a special degree. Of course, a major goal is enabling students to critically assess the validity of research articles themselves, but also providing experience with available statistical software should be striven for. The already developed PC-seminar in biostatistics, serving as a framework for the current study, addresses these requirements. Also the medical licensure act 2004 for German physicians defined exactly those demands [5], [14]. The presented NaNa concept extended the well-established PC-seminar and included a self-collection of empirical study data with subsequent analysis in order to increase motivation among the participating students. By incorporating the students already in the data collection process it was expected that this also would have a positive effect on learning success. However, the results showed that there was no significant improvement with respect to both outcomes of the study. In fact, the control group even achieved higher cumulative scores with 113.8 points against 109.0 points in the NaNa group, which corresponds to a moderate effect size. Consequently, students of the control group on average got better grades (1.6 in the control group vs. 2.1 in the NaNa group). In light of the number of participants who have been investigated in the course of this study, it is not possible at that point to conclude that this is a systematic effect. A more comprehensive evaluation of the differences found in our pilot study would require to have larger studies providing more statistical evidence. However, based on mean cumulative score of 110 points (SD 8.9) during the past years (corresponds to an overall grade 2), a total sample size of N=160 students would be required to demonstrate a significant effect of the NaNa intervention assuming the students to improve their results to 114 points (overall grade 1.5) assuming a power of 80% and a two-sided type 1 error of 5%. This could be realized by either extending the observation on multiple semesters or by a multi-centric approach. Such a study, however, has to be scheduled as a cluster-randomized study in order to prevent possible differences of the involved universities [15].

Regarding the effect of the NaNa concept on student’s acceptance there was a slight tendency of the interventional group being superior. The overall rating of the seminar based on regular school grades revealed a higher median score in the NaNa group compared to the control group receiving the standard didactic approach. Likewise, students of the NaNa group answered more convincing to the question whether they would attend the seminar again. 

### Limitations

It was not possible to implement our study, as initially planned, as a cluster-randomized study allocating the four seminar groups to either interventions. This was primarily due to organizational reasons (class schedule), but especially because of the limited capacity of the available PC-labs. Both seminar groups taking place on Tuesday provide 24 working places, whereas the seminar groups on Thursday and Friday have only 14 working places available. Randomization would have had the potential drawback that accidently both smaller seminar groups were chosen to apply the NaNa concept. Thus, the theoretically available outcome variables for the interventional group would have been decreased from potentially 48 to 28 values. In light of the fact that the cumulative sum of points following the standard educational concept was constant over the last years, we thought it was rather acceptable to have only 28 values for the control group. Moreover, the comparative analyses summarized in Table 1 (Tab. 1) showed no difference between both collectives, except for the distribution of semesters. This difference is not surprising, since students of the 6^th^ semester were not able to attend the PC-seminar on Thursday and Friday (both control groups) because of their specific class schedule. 

Overall, the sample size in this pilot study is certainly falling short of deriving valid statements from the conducted analyses. Especially the results of the control group referring to the acceptance of the course are not representative relying on only 26 students. However, the control groups’ results regarding learning success are indeed valid since they perfectly match with the cumulative sum of points from earlier semesters. 

With respect to the small effects found in this study it is important to note that the grades in biostatistics have been on a high level in the past years. Therefore, it was clear at the outset that distinct improvements in learning success can hardly be achieved. As noted above, a distinctively larger sample would be required in order to demonstrate an improvement in learning success when applying the NaNa concept. Moreover, all study participants attended the data collection in order to get a preferable large data set. As a result of this, however, also students of the control group have experienced activating elements. 

## Conclusion

The present intervention study was not able to confirm the predefined research hypotheses assuming the activating didactic concept to be superior to the standard approach. As expected, it was only found that motivation and qualitative evaluation of the seminar showed a slight tendency of being increased in the NaNa group. However, more extensive data are required to validly assess the effect of self-assessment as an activating teaching method on learning success and acceptance of the subject biostatistics. Future studies may include additional outcome measures, e.g. motivation score tools [16] enabling a more comprehensive evaluation of the teaching concept. Taking into account all the arguments raised before and in spite of the unverifiable impact of the applied intervention on learning success, education in biostatistics should perpetuate implementing practical examples in order to give students an understanding of the subject’s relevance.

## Competing interests

This study was funded by the AG Lehrforschung of the Medical Faculty, Ulm University. There are no conflicts of interest for any of the authors. 

## Figures and Tables

**Table 1:**
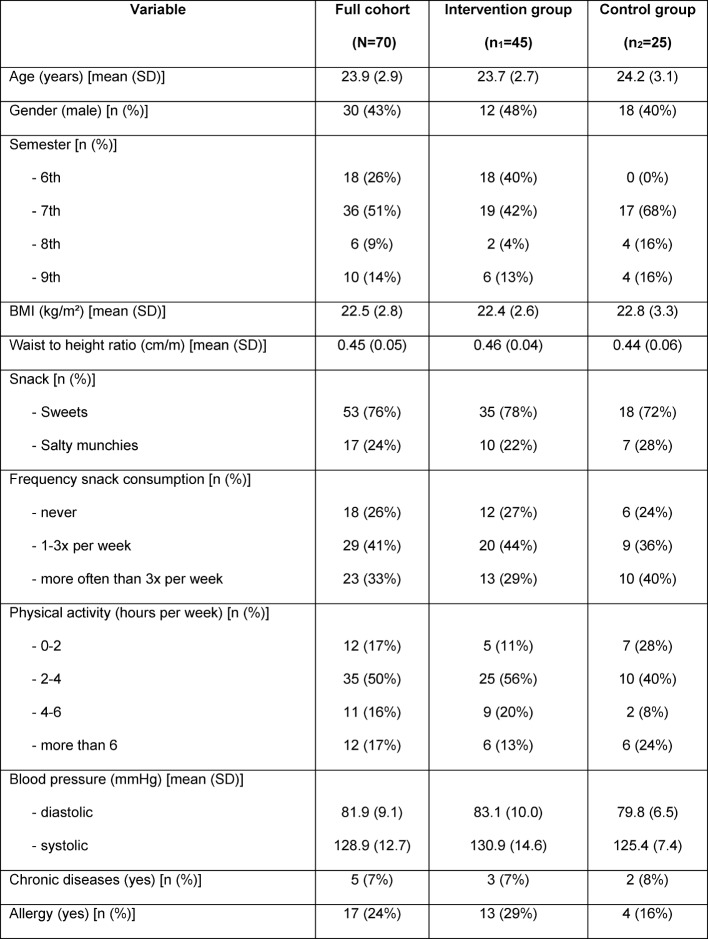
Sample characteristics
